# Respiratory epithelial cell types, states and fates in the era of single-cell RNA-sequencing

**DOI:** 10.1042/BCJ20220572

**Published:** 2023-07-06

**Authors:** Oleksandr Dudchenko, Jose Ordovas-Montanes, Colin D. Bingle

**Affiliations:** 1 Department of Infection, Immunity and Cardiovascular Disease, The Medical School, University of Sheffield, Sheffield, South Yorkshire, U.K.; 2 Division of Gastroenterology, Hepatology and Nutrition, Boston Children's Hospital, Boston, MA, U.S.A.; 3 Programme in Immunology, Harvard Medical School, Boston, MA, U.S.A.

**Keywords:** cell biology, cell fate, respiratory epithelium, scRNA-seq

## Abstract

Standalone and consortia-led single-cell atlases of healthy and diseased human airways generated with single-cell RNA-sequencing (scRNA-seq) have ushered in a new era in respiratory research. Numerous discoveries, including the pulmonary ionocyte, potentially novel cell fates, and a diversity of cell states among common and rare epithelial cell types have highlighted the extent of cellular heterogeneity and plasticity in the respiratory tract. scRNA-seq has also played a pivotal role in our understanding of host–virus interactions in coronavirus disease 2019 (COVID-19). However, as our ability to generate large quantities of scRNA-seq data increases, along with a growing number of scRNA-seq protocols and data analysis methods, new challenges related to the contextualisation and downstream applications of insights are arising. Here, we review the fundamental concept of cellular identity from the perspective of single-cell transcriptomics in the respiratory context, drawing attention to the need to generate reference annotations and to standardise the terminology used in literature. Findings about airway epithelial cell types, states and fates obtained from scRNA-seq experiments are compared and contrasted with information accumulated through the use of conventional methods. This review attempts to discuss major opportunities and to outline some of the key limitations of the modern-day scRNA-seq that need to be addressed to enable efficient and meaningful integration of scRNA-seq data from different platforms and studies, with each other as well as with data from other high-throughput sequencing-based genomic, transcriptomic and epigenetic analyses.

## Introduction

The ability to study gene expression at a transcriptome-wide scale in organisms, tissues and single cells was historically limited by the lack of quantitative, high-throughput strategies. Only at the turn of this century has the field of transcriptomics started realising its true potential, driven by the development of two revolutionary technologies [1,2]. Initially, transition from techniques such as Northern blotting and quantitative PCR to oligonucleotide microarrays increased the number of genes profiled per assay by several orders of magnitude [3,4], whereas roughly a decade later, RNA-sequencing (RNA-seq) not only enabled detection of novel transcript isoforms, but also allowed absolute quantification of mRNA species in a sample [5]. The issue of averaged gene expression values for the studied population of cells, which concealed cellular heterogeneity in both microarray and bulk RNA-seq data, was subsequently addressed by the development of RNA-seq at a single-cell resolution (scRNA-seq) [6–10].

Advances in single-cell isolation methods, which increased the throughput to thousands of cells per experiment [11], and reduction in cost per sequenced cell from around 10 USD [9] to approximately 0.5–1 USD on 10× Genomics Chromium platform [12], contributed to the global adoption of scRNA-seq over recent years. Increased need and funding for respiratory research during the coronavirus disease 2019 (COVID-19) pandemic further accelerated the implementation of scRNA-seq in the respiratory field [13], effectively positioning it at the forefront of developments in scRNA-seq and rendering the airways one of the most profiled systems in the human body. In addition to facilitating identification of cell types that are infected by severe acute respiratory syndrome coronavirus clade 2 (SARS-CoV-2) [14,15] and improving the understanding of factors affecting the outcome of SARS-CoV-2 infection [16], concurrent applications of scRNA-seq in homeostasis and disease have also led to a discovery of a novel epithelial cell type termed ionocyte [17,18], yielded new insights into the remodelling and dysfunction of the airway epithelium in smokers [19,20], individuals with chronic rhinosinusitis [21], asthma [22,23], idiopathic pulmonary fibrosis [24,25] and cystic fibrosis (CF) [26]. Furthermore, scRNA-seq has enabled creation of cell atlases of the healthy human lung by individual research groups [27–29] and by consortia. For instance, the first iteration of the LungMAP Single-Cell Reference atlas of the human lung, totalling almost 350 000 single-cell transcriptomes, was released in May 2022 [30]. On the initiative of the discovAIR consortium, the core version of the Human Lung Cell Atlas (HLCA) was also launched in early 2022, incorporating transcriptomic data from nearly 600 000 individual cells [31]. In June 2023, the release of the extended version of HLCA signified one of the largest consortia-led attempts to integrate and harmonise scRNA-seq and single-nuclei RNA-seq data from more than 2.4 million cells from 49 individual datasets [32]. scRNA-seq data accessibility through atlas-associated and standalone online tools, such as CZ CELLxGENE web application and UCSC cell browser, and data integration into the existing Lung Gene Expression Analysis web portal [33] provide user-friendly interfaces for exploration of complex scRNA-seq data and their interpretation along with results from other ‘omic’ and conventional techniques for gene expression analysis, respectively.

Since major findings attributable to the use of scRNA-seq in the respiratory field have already been extensively reviewed [34–37], this review attempts to showcase key developments in scRNA-seq methodology and to elaborate on some of the fundamental challenges arising from differences in experimental designs and computational approaches that need to be explicitly addressed when interpreting and contextualising scRNA-seq data. For instance, two most common analyses scRNA-seq data are subjected to — categorisation of cells into distinct groups and positioning of cells along the likely differentiation axes on the basis of similarity or dissimilarity between levels of select transcripts, raise several questions. First, to which extent is a transcriptomic snapshot on its own sufficient for defining a cell type? The absence of a consensus definition of a cell type [32,38] and of comprehensive models that consolidate various aspects of cellular identity [39] further complicate matters. Second, given the stochasticity of gene expression [40] as well as biological and technical noise inherent to scRNA-seq [41], thresholds used to identify the proportion of transcriptional variation between individual cells that is both true and biologically meaningful play a pivotal role in interpretation of scRNA-seq results. Third, with various types of sequencing platforms available and a rapidly growing number of data analysis tools, the reproducibility of the discoveries and phenotype-associated outcomes of scRNA-seq studies needs to be critically evaluated. Confounding factors, on top of those introduced by specific scRNA-seq protocols, include interindividual differences, variability in sample acquisition and processing, and distinct tissue culture and differentiation methods used in *in vitro* studies. Although these and other challenges are discussed here in the respiratory context, our insights will be applicable to scRNA-seq conducted in additional tissue systems. Due to the complexity of the respiratory tract, we mainly focus on the ways scRNA-seq has influenced the knowledge about heterogeneity and plasticity of epithelial cells in the lower airways of humans, from trachea to alveoli, with brief context added from studies of upper airways and model organisms.

## scRNA-seq workflow

**Figure 1.**
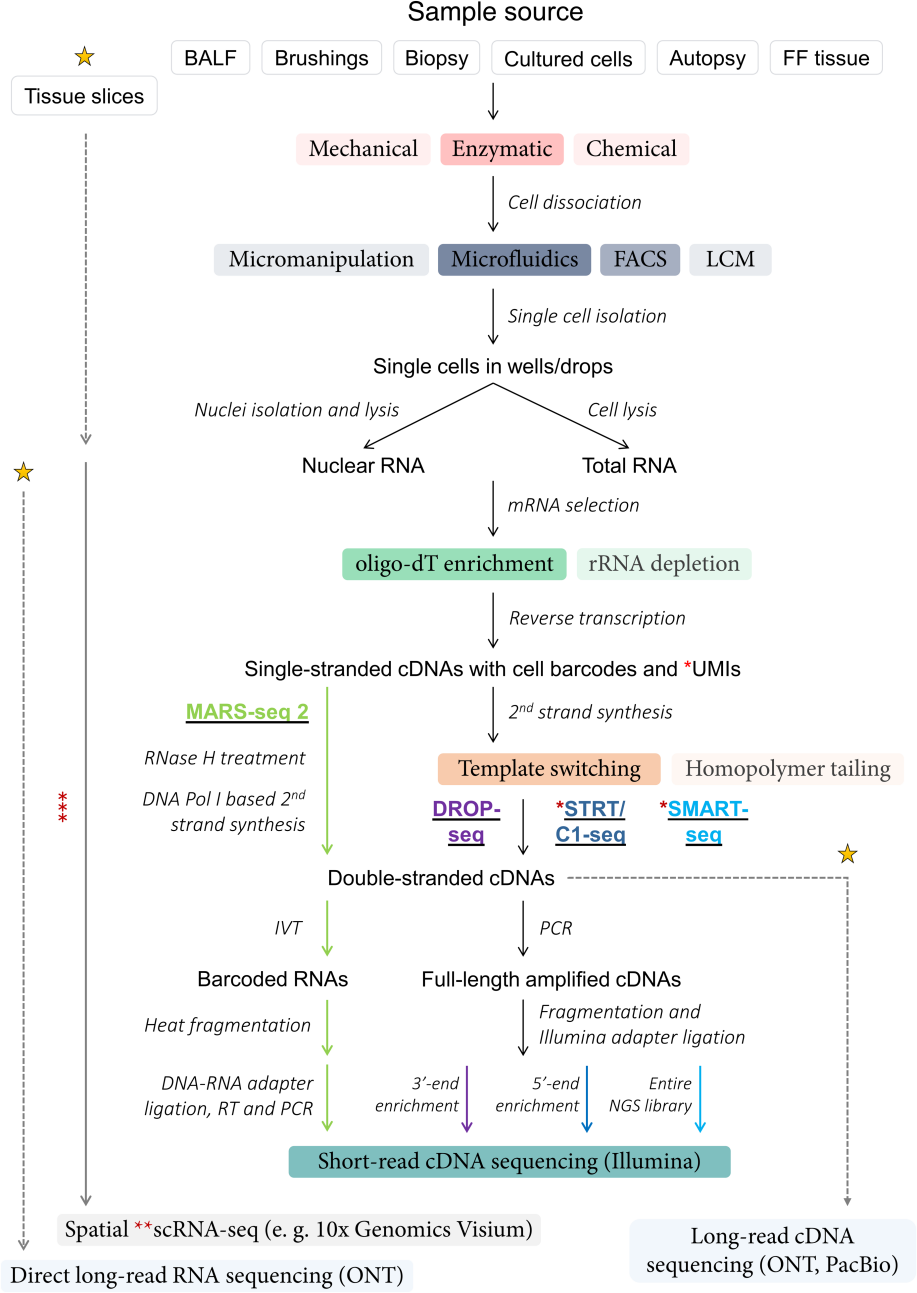
Process of sc-RNA sequencing. Summary of shared sample processing steps and simplified final stages of next generation sequencing (NGS) library preparation in established scRNA-seq protocols (MARS-seq 2 — light green font and arrows; DROP-seq — purple font and arrow; STRT/C1-seq — navy font and arrow; SMART-seq — light blue font and arrow). *Key*: black arrows — shared steps; coloured boxes — methods (colour intensity corresponds to relative frequency of method usage in scRNA-seq protocols); italicised text — experimental stages; yellow stars — emerging and/or future scRNA-seq technologies (details not shown); dotted grey arrows — stages of conventional protocols that may be (partially) bypassed with emerging and/or future scRNA-seq technologies; * — unique molecular identifiers (UMIs) were not incorporated into complementary DNAs (cDNAs) in the original STRT/C1 sequencing protocol, SMART-seq v1 and v2 protocols; ** — majority of currently available spatial transcriptomic technologies provide regional rather than single-cell resolution; ***(solid grey arrow) — methodology for emerging spatial transcriptomic techniques not shown; FACS — fluorescence-activated cell sorting; LCM — laser capture microdissection; FF — formalin-fixed; BALF — bronchoalveolar lavage fluid (including endotracheal aspirate); IVT — *in vitro* transcription; ONT — Oxford Nanopore; PacBio — Pacific Biosciences.

Overcoming technical bottlenecks at the level of single-cell capture and early multiplexing [11], numerous scRNA-seq techniques have been developed and revised since the publication of the pioneering paper by Tang et al. [6]. With timeline figures juxtaposing years of introduction and throughput of exhaustive lists of scRNA-seq techniques generated in other publications [11,42] and given that most protocols share core principles (Figure 1), one way of simplifying categorisation of a large variety of diverse scRNA-seq methods can be based on the extent of transcript coverage with short-read Illumina complementary DNA (cDNA) sequencing — full-length, 5′- and 3′-end. One of the most frequently used plate-based methods from the full-length group is SMART-seq [7,43,44], with the latest iteration being SMART-seq 3. SMART-seq-based scRNA-seq experiments are usually conducted on a relatively small number of cells, ranging from a few hundreds to several thousands, which are sequenced at a depth of up to 1–2 M reads per cell. Early 5′-end scRNA-seq methods included STRT-seq [45] and STRT/C1 [46], while common 3′-end methods encompass CEL-seq [47,48], MARS-seq [49,50], Cyto-seq [51] and DROP-seq [8] (Figure 1). Droplet-based 3′-end scRNA-seq platforms developed in academic settings or in industry, such as DROP-seq [8] and 10× Genomics Chromium, respectively, generally support higher experimental throughput, but often at the expense of sequencing depth and transcript coverage, resulting in less transcriptomic detail being captured per cell. With thorough descriptions of methodologies provided in the original papers and compared in dedicated reviews [52–54], it is worth highlighting several key features of modern-day scRNA-seq protocols.

First, incorporation of unique molecular identifier (UMI) sequences into the cDNA molecules, which is a step in most 3′-end scRNA-seq methods [55] and in SMART-seq 3 [44], enables absolute quantification of transcripts, identification and elimination of PCR duplicates [56,57]. RNA ‘spike-in’ molecules, in turn, are widely used for normalisation during data analysis [41,58] and, if modified via addition of internal UMIs, can improve the accuracy of RNA quantification in both droplet- and plate-based scRNA-seq methods [59]. Lastly, in light of the improving accuracy of long-read sequencing technologies from Oxford Nanopore (ONT) and Pacific Biosciences (PacBio) [60], one can envision the increase in their adoption for scRNA-seq in the near future [61] (Figure 1) due to their ability to identify novel and differentially expressed transcript isoforms [62,63]. Introduction of the spatial context into scRNA-seq is also promising [64] (Figure 1), but current commercial platforms, such as 10× Genomics Visium, provide regional rather than single-cell resolution when it comes to *in situ* mRNA capture, barcoding and sequencing.

**Figure 2.**
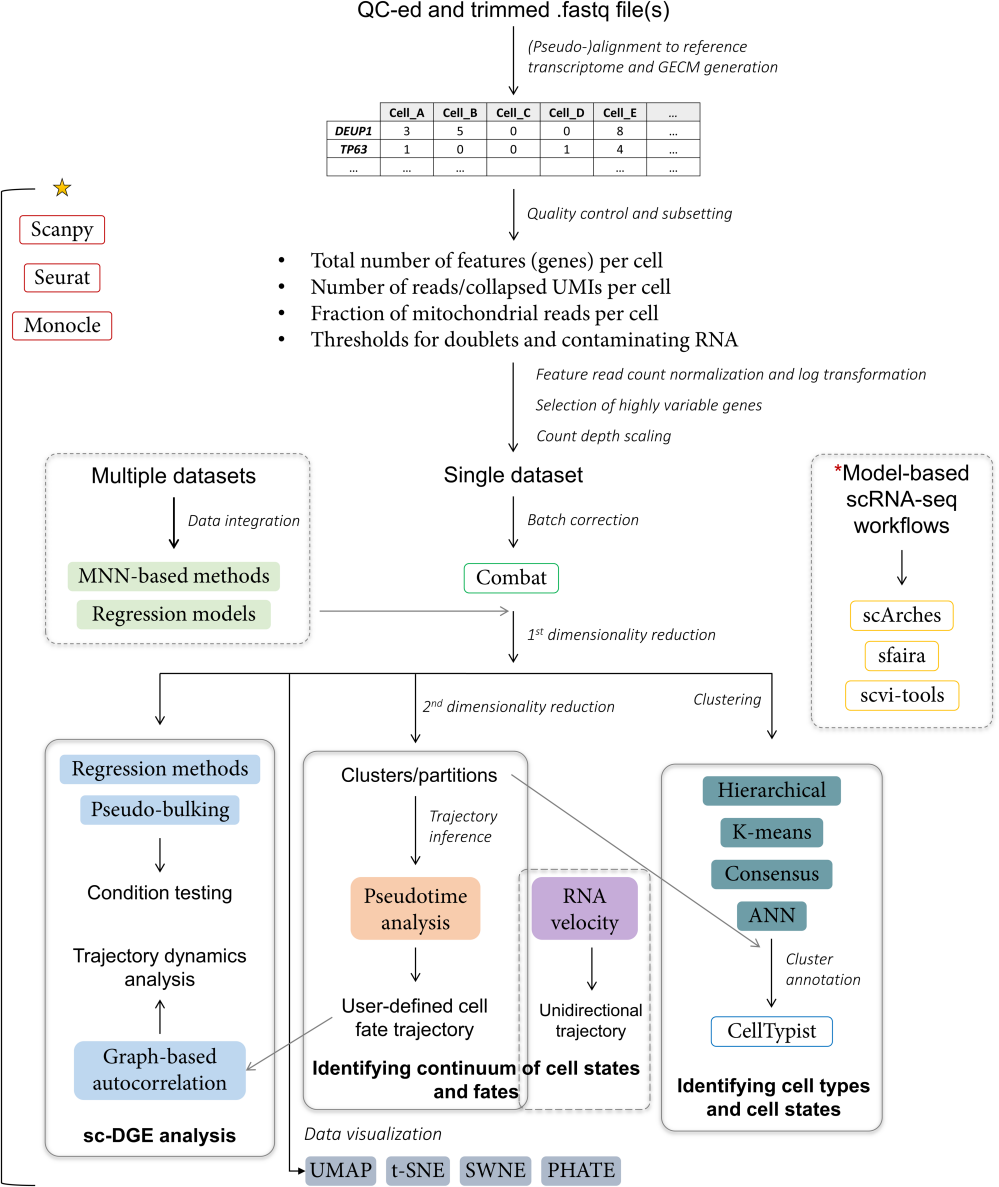
Analysis of sc-RNA-seq data. Summary of key stages in short-read scRNA-seq data analysis with examples of general methods (coloured, filled boxes), common bioinformatic tools (boxes with coloured outline) and key downstream applications (boxes with solid grey outline). Following standard Illumina read quality control (QC) and adapter trimming, paired or single-end reads are mapped to the reference genome or transcriptome and gene expression counts matrix (GECM) is generated. Afterwards, potentially non-viable cells, cell doublets and contaminating RNAs [74,75] can be filtered out *in silico*. Read counts for each cell passing set criteria are then log-normalised. Having selected highly variable genes for marker identification and optionally, having regressed cell cycle or other classes of genes, log-normalized counts are scaled, giving each gene equal weight in downstream analyses. If a dataset is obtained from an experiment conducted in multiple batches, batch correction can be applied [76]. Analysis of datasets from experiments with different designs will require data integration. Subsequently, the first dimensionality reduction is performed, typically with principal component analysis (PCA). Resulting data can then be visualised; used for single-cell differential gene expression (sc-DGE) analysis; undergo further dimensionality reduction and used for inferring developmental or condition-driven trajectories; clustered and annotated to identify cell states and cell types [77]. *Key*: yellow star — majority of the highlighted analysis steps can be performed using three listed software packages [66–68]; * — alternatively, data analysis workflow based on reference models generated with machine learning algorithms can be selected [78–80], particularly when integrating multiple datasets from the same tissue; UMI — unique molecular identifier; ANN — approximate nearest neighbours; UMAP — uniform manifold approximation and projection; PHATE — potential of heat diffusion for affinity-based transition embedding; SWNE — similarity-weighted non-negative embedding; t-SNE — t-stochastic neighbour embedding.

Similarly to sample preparation and sequencing protocols, many tools for analysis of scRNA-seq data were developed by leveraging insights from bulk RNA-seq [65]. Currently, researchers’ options range from proprietary software packages (e.g. 10× Genomics Cell Ranger) to open-source, integrated pipelines such as Seurat [66], Monocle [67], Scanpy [68] and more than 1500 individual scRNA-seq tools [69], which can be mixed and matched to create a highly customised data analysis strategy. With in-depth coverage of general and scRNA-seq-specific bioinformatics being beyond the scope of this review, only the key stages of scRNA-seq data pre-processing, main downstream analyses, common statistical methods and software packages are briefly summarised here (Figure 2). For conceptual overview of each stage and step by step practical guidance, reviews by Wu and Zhang [65], and Luecken and Theis [41], respectively, are recommended.

Although barriers of entry to scRNA-seq data analysis have been substantially lowered both in terms of technical requirements [70] and accessibility to individuals with minimal knowledge of programming, it remains a resource-intensive process, particularly when it comes to analysis and integration of datasets from tens or even hundreds of thousands of cells. Biological hypotheses and method awareness are required for evaluation, selection of filters and parameters at most stages of scRNA-seq data analysis, including quality control, data normalisation, dimensionality reduction and cell cluster annotation. Data re-analysis can also often be hindered by incomplete documentation of conducted bioinformatic analyses in literature. In other words, even if a pipeline or package names are specified in the methods section, re-running them on the same raw data may produce different results unless the exact parameters for each step, e.g. number of UMIs per viable cell or a cell (node) that serves as a starting point for trajectory inference, are provided by the authors. The impact of these factors as well as of the high degree of customisation, large number of zero expression values [71], variable performance of different clustering [72] and trajectory inference methods [73] will be elaborated on in further sections.

## Evolving definitions

### Cell types

**Figure 3.**
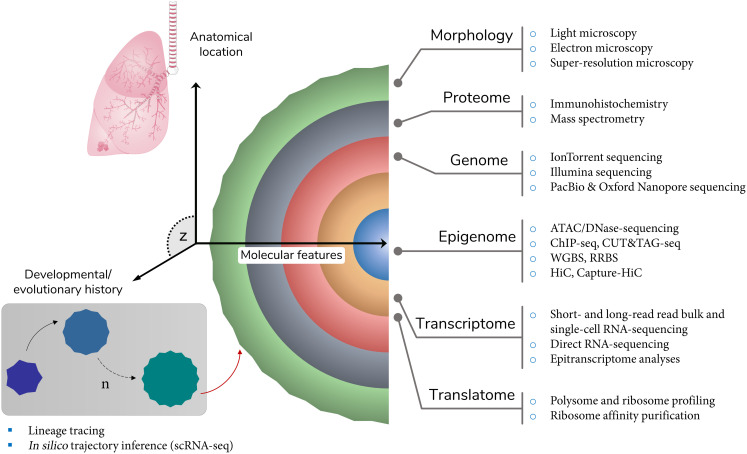
Features of cellular identity. Common features of cellular identity that can be used individually or collectively to define a cell type, with examples of conventional and ‘omics’ techniques that can be used to investigate each set of features. *Key*: z space — other sets of features (not shown) that are independent or derived from molecular and evolutionary parameters and can be incorporated into the definition of a cell type; red arrow — directional outcome of the sequence of evolutionary/developmental events that led to the current form of the cell; sc — single cell; seq — sequencing; PacBio — Pacific Biosciences; ATAC — assay for transposase accessible chromatin; ChIP — chromatin immunoprecipitation; WGBS — whole genome bisulphite sequencing; RBBS — reduced representation bisulphite sequencing; HiC — high-throughput chromosome conformation capture.

Parameter-specific categorisation of cells of multicellular organisms into types is one of the most fundamental concepts in biology [81]. Highlighting the multifaceted nature of this practice, field-specific perspectives on the definition of a ‘cell type’ across different branches of biological science are not uncommon [38]. Adhering to a more prevalent type of definition, Vickaryous and Hall [82] catalogued cell types only if similar cells in question occurred *in vivo*, were terminally differentiated as well as at the same stage of developmental history and cell cycle. While some of these assumptions hold true for currently accepted cell types, it is not uncommon for some of the cell types to diverge from them, particularly with respect to differentiation status. To exemplify, Tata and Rajagopal [83] demonstrated that upon injury murine club cells were able to dedifferentiate into basal epithelial cells. Looking beyond the classification systems based on resemblances in cellular morphology, marker gene expression etc., evolutionary definitions of a ‘cell type’ have also been proposed. For instance, according to Arendt et al. [84], cells can be deemed of the same ‘type’ if they are more evolutionarily related than other cells of the same organism. These examples hint at the difficulty of arriving at a consensus definition of a cell type. However, it is apparent that for the majority of cell types to be definitively recognised as such, not just a single or several properties, but rather — an ensemble of features across multiple sets of parameters constituting a cell (Figure 3), from commonality in the studied population [85] to epigenomes, should be identified, validated and integrated. On top of that, the variability between cells of the same type, which can be observed even at the intra-individual level due to intrinsic stochasticity of nearly all cellular processes, sampling effects or technique artefacts, and can be further amplified during scRNA-seq data analysis, should be factored in. Hence, an optimal consensus definition of a ‘cell type’ should stipulate both commonly used and acceptable number of cell identity features with corresponding thresholds of variance, thereby providing a tentative framework, while balancing potential for discovery and promoting data exploration.

Two of the most comprehensive standalone single-cell atlases of the healthy human lung to date were constructed by Deprez et al. [28] and Travaglini et al. [27], with transcriptomes of nearly 80 000 cells profiled by each group. Atlas generated by Deprez et al. [28] is mainly comprised of lung epithelial cells from dozens of different locations along the airway tree, whereas the latter atlas also contains large fractions of vascular and immune cell types from the lung. Among major findings of Travaglini et al. [27], a wide range of intra- and intercellular signalling pathways was elucidated at a greater resolution, expression of key lung-disease genes were localised to cell types and differences between gene expression patterns of human lung cell types and murine counterparts were highlighted. Importantly, the authors also increased the cell type count in the lung to 58 individual cell types. This figure includes 14 newly reported cell types, many of which belong to stromal, endothelial and immune cell lineages that are not discussed here. As for the epithelial cells, Travaglini et al. [27] listed populations of ‘proximal ciliated’, ‘proximal basal’ and ‘alveolar type II signalling’ cells as ‘novel’ cell types. Deprez et al. [28], in turn, described two ‘novel’ cell types — ‘multiciliating-goblet’ and ‘undefined rare’ cells. Whether and to what extent these cell populations, alongside ‘proliferating/cycling basal’ and ‘differentiating basal’ cells, can be classified as ‘novel’ and/or as ‘cell types’ requires large-scale integrative efforts for many reasons outlined in the next section of this review. However, inclusion of ionocytes into lists of epithelial cell types that are found in the human lungs has been widely accepted and can be observed in all major publications on this topic.

Discovered concurrently by Montoro et al. [17] and Plasschaert et al. [18], pulmonary ionocytes can arguably be considered the first novel airway epithelial cell type identified using scRNA-seq by capitalising on one of the main strengths of the technique — the ability to detect rare cells with unique gene expression signatures. Distinctive cell clusters, which were highly enriched in transcripts of genes encoding a plasma membrane chloride ion transporter, cystic fibrosis trans-membrane conductance regulator (CFTR), a transcription factor required for *CFTR* expression, FOXI1, subunits of V-type ATPase, ATP6V1C2 and ATP6V0D2 and other proteins conducive to the regulation of properties of the airway surface liquid, were first detected in mouse tracheal and human bronchial tissues as well as among human bronchial epithelial cells (HBECs) differentiated at the air–liquid interface (ALI) [17,18]*.* With the exception of *CFTR*, similar gene expression patterns were previously reported for intercalating non-ciliated cells in the skin of *Xenopus laevis* [86] and for ionocytes in the gills of fish [87], but had not been reported in the mammalian respiratory tract. The presence of ionocytes was also confirmed using immunostaining for the aforementioned markers by Montoro et al. [17] and Plasschaert et al. [18], and subsequently — with scRNA-seq, immunostaining and/or fluorescence *in situ* hybridisation in more recent studies of both healthy [20,27,28] and diseased human airways [19,22,23,26].

**Figure 4.**
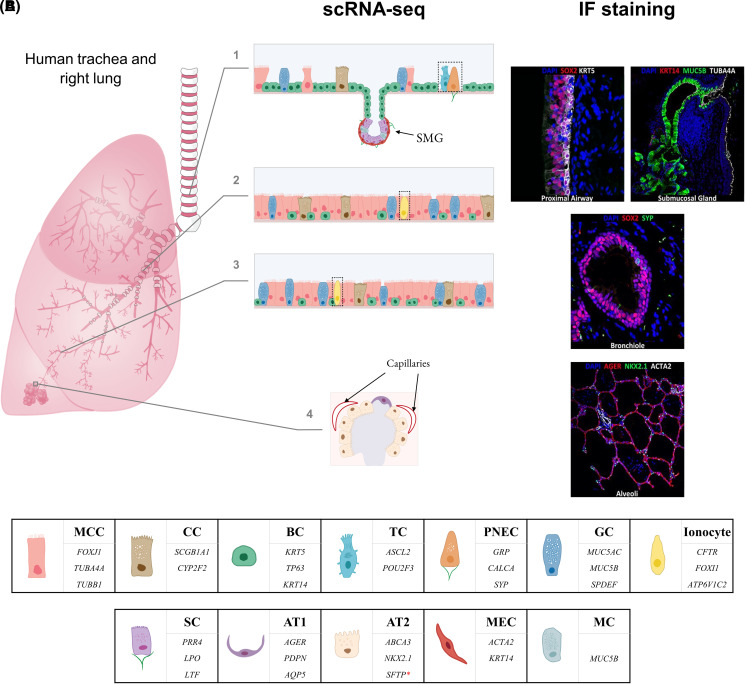
Cell populations in the human lower airways. (**A**) Epithelial cell landscape of the human lower airways based on select scRNA-seq data and immunofluorescence (IF) staining (undefined and novel ‘cell types’ that were either identified only in a single study or may better fit the description of ‘cell states’ or ‘subtypes’ of canonical epithelial cell types are not shown; where applicable, fractions of author-defined subpopulations of a canonical cell type were added up to represent each canonical cell type as a single category); 1 — trachea (tracheal cell type fractions roughly estimated from Deprez et al. [28], Figure 1F, tracheal biopsy stacked column; number of displayed cells (*n*) = 50); 2 — bronchi (bronchial cell type fractions as provided by Okuda et al*.* [20], Figure 1C; *n* = 37); 3 — bronchioles (bronchiolar cell type fractions as provided by Okuda et al. [20], Figure 1C; *n* = 38); 4 — alveoli (alveolar cell type fractions roughly estimated from Vieira Braga et al. [22], Figure 1D, parenchyma pie chart, *n* = 10; small fraction of identified multiciliated cells is not shown). IF images taken with permission from LungImage, Cincinnati Children's Hospital Medical Center. (**B**) Some of the main marker genes of canonical lung epithelial cell types [27,36]. *Key*: black dotted outline — cell types constitute less than 1% of the total sampled cell population but were included for illustration purposes; *SOX2* — marker of tracheal, bronchial and bronchiolar epithelial cells; BC — basal cell; GC — goblet cell; MC — mucous cells; MCC — multiciliated cell; SMG — submucosal gland (not shown in bronchi); CC — club (Clara) cell; TC — tuft (brush) cell; PNEC — pulmonary neuroendocrine cell (green lines — nerves); GC — goblet cell; AT1 — alveolar type 1 cell; AT2 — alveolar type 2 cell; MEC — myoepithelial cell; MC — mucous cell; SC — serous cell (green lines — nerves); SFTP* — *SFTPB*, *SFTPC*, *SFTPD*.

Provided sufficient power, in terms of the number of profiled cells, is achieved in a given scRNA-seq study, expression data are often used not only to determine the heterogeneity and plasticity of common and rare epithelial cell types in different sections of the respiratory tract, but also to illustrate their relative tissue distributions. For example, in some publications, relative frequencies of cell types are derived by dividing the number of cells mapping to a particular cluster by the total number of cells that were annotated from sequenced samples [22,88,89]. These may not always correlate with observations from conventional histological analyses, as illustrated by a relatively low abundance of multiciliated cells (MCCs) in the tracheal section of the proximal airway epithelium identified with scRNA-seq in contrast with immunofluorescence staining (Figure 4A). Nonetheless, such type of comparison is vital for highlighting potential challenges with cell dissociation protocols and other experimental factors that may lead to variable efficiencies in survival, capture and sequencing of particular cell types.

### Cell states

**Figure 5.**
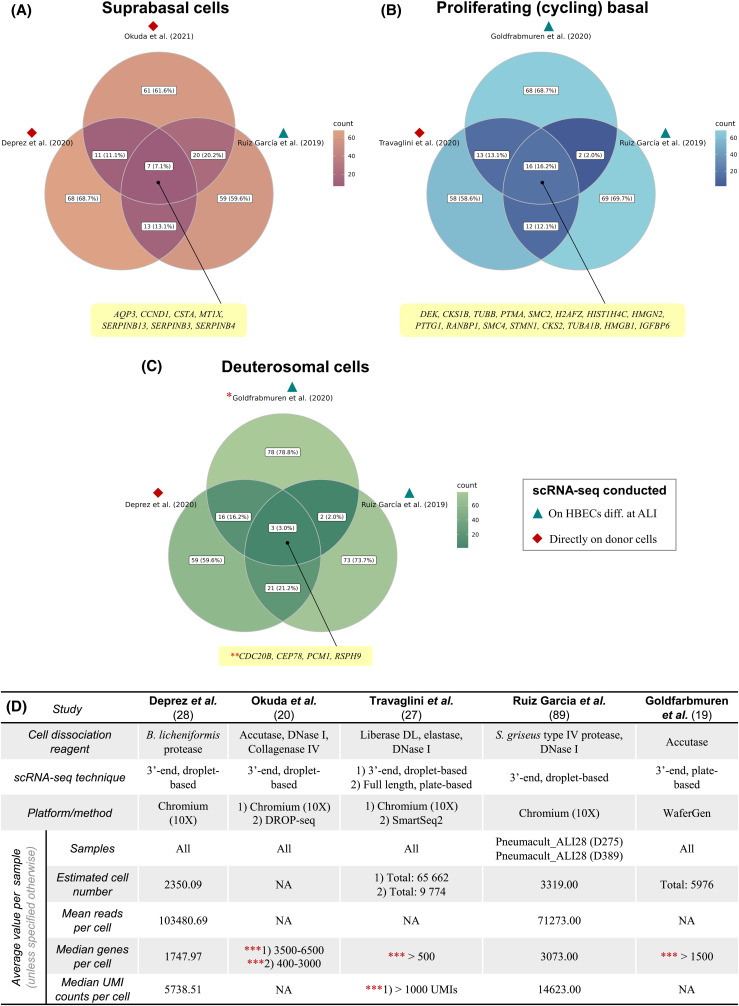
Correlations of gene expression profiles in specific cell populations. Extent of the overlap in gene expression between populations of suprabasal (**A**), proliferating basal (**B**) and deuterosomal (**C**) cells among top 99 marker genes (based on positive log fold change values) identified in multiple scRNA-seq studies, with metadata available on these cell populations provided for each paper (**D**). *Key*: (**A**) (suprabasal cells): Okuda et al. [20] — suprabasal cluster visualised on https://cells.ucsc.edu/?ds=lung-airway+boucher-epithelium; Deprez et al. [28] — Supplementary table E7; Ruiz García et al. [89] — Supplementary table S1; (**B**) (proliferating basal cells): Travaglini et al. [27] — Supplementary table 4; Goldfarbmuren et al. [19] — ‘Source data table 28 for Figure 5b’ in the original paper; Ruiz García et al. [89] — Supplementary table S4; (**C**) (deuterosomal cells): Deprez et al. [28] — Supplementary table E7; Goldfarbmuren et al. [19] — ‘Source data table 28 for Figure 5b’ in the original paper; Ruiz García et al. [89] — Supplementary table S4; * — cell population was referred to by authors [19] as ‘*FOXN4* early ciliating’; ** — expression of *CDC20B* in deuterosomal cells was reported by Deprez et al*.* [28] and Goldfarbmuren et al. [19], while Ruiz García et al. [89] only reported expression of *CDC20Bshort*; *** — only cells with specified number of genes or unique molecular identifiers (UMIs) were used in downstream scRNA-seq analyses; HBEC — human bronchial epithelial cells; diff. — differentiated; ALI — air liquid interface. Figure produced with R package *ggVennDiagram* [90].

Identification of canonical cell types via annotation of automatically generated or pre-determined number of clusters, which can be followed by subdivision of select clusters into smaller groups based on the expression levels of several to a few dozens of genes, commonly referred to as tiered clustering, is a recurring theme in a majority of large-scale scRNA-seq studies in the respiratory field. In some papers, author-defined subpopulations of established cell types are numbered, e.g. ciliated 1, ciliated 2, ciliated 3 [20], whereas in others — they are given names that attempt to characterise their general transcriptome states, e.g. proliferating, differentiating and proteasomal basal cells [19], anatomical locations, e.g. proximal and distal basal cells [27] or morphological appearance, e.g. hillock club and basal cells [17]. In addition to potential confusion that may be caused by subpopulation names that vary from publication to publication, especially when a simple numbering strategy is chosen, such approach for classification of cells is frequently accompanied by inconsistent use of terms that describe aspects of cellular identity or by lack of reproducibility. For instance, a subpopulation of MCCs, also known as ‘deuterosomal cells’, which is enriched in transcripts of early ciliogenesis genes (e.g. *CDC20B*, *DEUP1*, *FOXN4*), is simultaneously categorised as a subtype of MCCs [89], recognised as an independent cell type [28] and is not even found in the first place [27]. Further exacerbating the issue, a comparison of expression profiles of the aforementioned populations and two other subpopulations identified in three different studies, yields mixed results (Figure 5), with mere 4–16 markers being shared out of top 99 differentially expressed genes (DEGs). This number of DEGs was selected because positive log fold change values were available only for 99 genes in two studies for the suprabasal population [20,89]. It is worth noting that a more thorough comparison of clusters between studies would require complete transcriptome integration or correlation analyses, and any conclusions based on whether a particular gene is transcribed or not do not fully reflect observed variation in gene expression between cells. This superficial analysis, however, indicates that only a few genes, which are not necessarily at the top of the DEG list, are identified as shared markers of subpopulations of cells with similar or identical names that were detected in multiple scRNA-seq experiments. Interestingly, sample source in selected scRNA-seq studies and cell populations appears to have little to no impact. For example, proliferating (cycling) basal cells identified among HBECs differentiated at the ALI [19,89] bear greater individual resemblance the same cell population identified in scRNA-seq conducted directly on donor cells [27] than to each other in terms of the number of shared top 99 DEGs (Figure 5B). To what extent this observation and general discrepancies between compared cell populations are affected by variability in cell throughput or sequencing depth remains unclear as key quality metrics were only fully specified by two out of five papers (Figure 5D).

Although the requirement for consensus nomenclature, when it comes to differentiating between ‘cell type’, ‘cell state’ and similar terms that are often used interchangeably, has been highlighted before [36,91], there are multiple ways in which it can be satisfied. Under ideal circumstances, reference values and their scope outlined in publications such as meta-analyses and reviews should provide sufficient guidance for researchers on how to improve scRNA-seq data interpretation and gradually standardise the use of key definitions. In practice, experiment- or hypothesis-dependent aspects that may not be covered by such guidance as well as the absence of certain data quality and processing criteria, which can be set by publishers, reviewers, peers etc., may not result in widespread adoption of the guidance. An alternative and more likely solution, given the growing number and size of large scRNA-seq cell atlases, can be the use of reference cell type annotations provided by consortia, which in the respiratory research field are mainly represented by HLCA [31,32] and LungMAP [30]. For example, in the recently released extended HLCA, a new cell identity reference framework was proposed following re-annotation of clusters from integrated datasets and input from experts in the respiratory research field [32]. If implemented as an additional quality control (QC) step, e.g. the number and identities of clusters from scRNA-seq experiments can be cross-checked against a known number of cell types, their relative proportions and expression profiles identified with scRNA-seq within the tissue of interest, such reference annotations will be of great value. The long-term success of this approach, however, will depend on the capacity of consortia to incorporate more datasets as they are published, to regularly evaluate the toolkit available for data analysis and integration, and if necessary re-run pipelines, as well as to set and dynamically adjust, with minimum bias from expert panels, the fundamental criteria for defining ‘cell types’, ‘cell states’ etc. For instance, given that all cells from a particular lineage share expression of several marker genes, e.g. transcription factor SOX2 is expressed by all epithelial cells in the proximal airways [92], a threshold needs to be established for the number of genes that need to be expressed exclusively in a subpopulation of a canonical cell type for it to be recognised as a novel cell type rather than a more specialised subtype or a transient state induced by a particular cellular or experimental condition. In addition, the relative weights of parameters such as the extent of differential expression for genes expressed in both of the compared cell populations and the frequency threshold of these populations among individuals will need to be provided. Lastly, datasets from spatial transcriptomics and other high-throughput single-cell analyses will introduce additional layers of complexity, but their incorporation into cell atlases is expected to increase the confidence in cellular identities determined with scRNA-seq.

### Cell fates

Since the first use of microscopy-based direct observation methods on organisms with determinate patterns of cell fate, the primary toolkit for studying the history of cell divisions from an ancestor cell to its terminally differentiated descendants, prospective lineage-tracing, has advanced from techniques involving tissue transplantation, usage of tracing dyes and transgenic fluorescent reporters [93] to high-throughput approaches facilitated by next generation sequencing (NGS). For instance, genomes of each cell in a population can now be uniquely barcoded via transgenic integration, *in vivo* recombination or live editing, thereby enabling reconstruction of cell lineages following DNA or RNA-sequencing [94–96].

Notwithstanding the rapid progress in prospective analyses, none of them can be conducted in humans and until recently, most of the information about human cell lineages has been either inferred from genetic lineage-tracing experiments in model organisms or obtained from *in vitro* differentiation of primary cells and retrospective lineage-tracing studies, in which phylogenetic lineages of cells are reconstructed on the basis of somatic mutations [97], particularly in microsatellite regions [9]. With the advent of scRNA-seq, not only a novel method of clonal tracking based on mutations and chromatin accessibility changes of mitochondrial DNA has been proposed [98], but also several *in silico* approaches, known as trajectory inference methods, have been developed for the determination of the relative developmental history of cells in a studied population [73] (Figure 2). As for the principles of key methods, pseudotime analysis attempts to replicate the temporal aspect of differentiation or any event resulting in changes in cell states or types by positioning cells or clusters along a pseudo-temporal axis or axes based on the degree of similarity in gene expression [67,73]. It should be noted, however, that pseudotime is not a chronological or unidirectional measure and akin to many other trajectory analysis tools, it is only intended to provide a framework for inferring directionality [99] on the basis of a biological hypothesis. A more recent type of analysis — RNA velocity, which is often classified as a trajectory inference method despite being an inherently unidirectional measure, identifies transitional cell states and positions them along a temporal axis based on the ratios of spliced to unspliced mRNAs [100]. Efficient integration of these two methods [101] and coupling of scRNA-seq with a dual fluorescent reporter-gene based system that can provide an absolute temporal scale of differentiation have also been reported [102].

**Figure 6.**
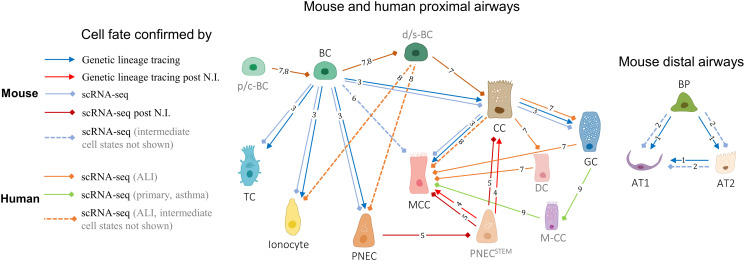
Cellular relationships in the lower airways. Simplified tree of the epithelial cell fates in the lower airways of mice and humans based on the recent lineage-tracing and scRNA-seq data (submucosal gland cell types and some de-differentiation pathways are not shown). *Key*: black labels — cell types; grey labels — putative cell states; N.I. — naphthalene-induced injury; p/c-BC — proliferating/cycling basal cell; BC — basal cell; d/s-BC — differentiating basal or suprabasal cell; BP — bipotent progenitor cell; AT1 — alveolar type 1 cell; AT2 — alveolar type 2 cell; CC — club cell; GC — goblet cell; DC — deuterosomal cell; M-CC — mucous ciliated cell; MCC — multiciliated cell; TC — tuft cell; TC-like — tuft-like cell; PNEC — pulmonary neuroendocrine cell; PNEC^STEM^ — pulmonary neuroendocrine stem cell. References: 1 — Desai et al. (2014) [104]; 2 — Treutlein et al. (2014) [105], 3 — Montoro et al. (2018) [17]; 4 — Song et al. (2012) [106]; 5 — Ouadah et al. (2019) [107]; 6 — Byrnes et al. (2022) [108]; 7 — Ruiz García et al. (2019) [89]; 8 — Goldfarbmuren et al. (2020) [19]; 9 — Vieira Braga et al. (2019) [22].

Epithelial cell lineages in healthy and injured airways of the most common small animal model of the human respiratory system, the house mouse [103], were mainly discovered using genetic lineage-tracing techniques [83] (Figure 6). In the distal mouse lung, alveolar type 1 and 2 epithelial cells were found to arise from a common bipotent progenitor, with the latter cell type being capable of regenerating the former [104], which was subsequently confirmed with scRNA-seq [105]. In a later study, Montoro et al. [17] performed both scRNA-seq of murine tracheal epithelial cells, in which common and rare epithelial cell types were identified, and *in vivo* genetic lineage-tracing providing temporal resolution of differentiation. Their results aligned with the existing knowledge about progenitors of canonical epithelial cell types and showed that populations of ionocytes, tuft and pulmonary neuroendocrine cells are likely derived from basal cells. The ability of mouse pulmonary neuroendocrine cells to replenish club and MCCs after naphthalene-induced injury, which was initially discovered using genetic lineage-tracing [106], was confirmed with scRNA-seq [107]. Byrnes et al. [108] also showed that differentiation of murine basal cells into MCCs may occur through an intermediate precursor cell in the absence of Notch signalling.

Results of both pseudotime and RNA velocity analyses in scRNA-seq studies of the human airways largely confirmed some of the proposed airway epithelial cell lineages [109] and led to discoveries of potentially novel developmental intermediates and differentiation routes [36], the presence of most of which still needs to be experimentally validated *in vivo* (Figure 6). For example, using nasal epithelial cells differentiated at the ALI, Ruiz García et al. [89] showed that differentiation of club cells into MCCs might occur through a developmental intermediate, the deuterosomal cell, and that goblet cells might act as direct precursors of MCCs. Goldfarbmuren et al. [19], in turn, used the same *in vitro* differentiation model established with tracheal epithelial cultures and identified distinct populations of ‘early’ and ‘later ciliating’ cells that might precede mature MCCs. As for scRNA-seq conducted directly on human bronchial biopsies, Vieira Braga et al. [22] detected a population of mucous ciliated cells, which were mainly present in asthmatic individuals and expressed markers of both mature multiciliated and goblet cells, that were proposed to eventually acquire a goblet cell fate.

## Limitations of scRNA-seq and good practice

**Figure 7.**
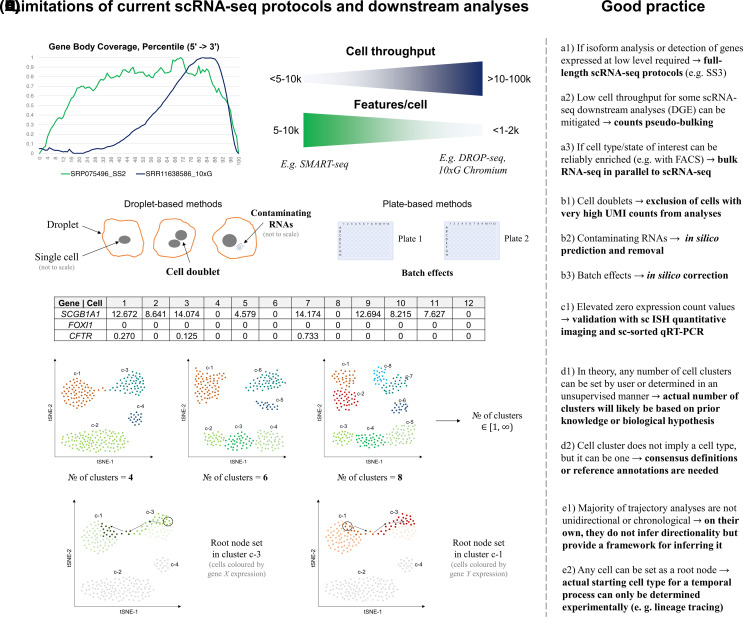
Limitations of sc-RNA-seq. Common limitations of current scRNA-seq protocols and downstream analyses alongside with the general strategies for avoiding pitfalls or mitigating known issues. *Key*: (**A**) (technical limitations of sequencing with examples of protocols) — in contrast with full-length scRNA-seq protocols such as SMART-seq2 and 3 (SS2 and SS3), 3′-end scRNA-seq protocols, e.g. DROP-seq and 10× Genomics Chromium (10×G), generally tend to prioritise cell throughput at the expense of gene body coverage and often, number of features (genes or unique molecular identifies) detected per cell (gene body coverage plots generated from data, which were downloaded using provided accession numbers, with RSeQC package, *geneBody_coverage2.py* [110]; (**B**) technical limitations of droplet- (e.g. DROP-seq) and plate-based scRNA-seq methods (e.g. SMART-seq); (**C**) (post transcript/gene quantification) — some scRNA-seq protocols may underestimate expression and cell type distribution of expression of certain genes whose transcripts are present at low copy number within the cells; (**D**) (examples of tiered clustering on a hypothetical scRNA dataset) — t-distributed stochastic neighbour embedding (t-SNE) plot, with cells coloured by cluster; (**E**) examples of a trajectory analysis on a hypothetical scRNA dataset (arrows indicate potential trajectory of development or differentiation); sc — single cell; ISH — *in situ* hybridisation; FACS — fluorescence-activated cell sorting; DGE — differential gene expression.

While duly acknowledging continuous improvements in throughput, sensitivity and ease of data manipulation, which has led to the current state of scRNA-seq (Figures 1 and 2), as well as appreciating insights obtained by analysing transcriptomes at a single-cell resolution, it is worth reiterating that scRNA-seq remains a highly nuanced technology that is still in the active stage of development. The quality of data from scRNA-seq experiments is affected by a wide range of factors, some of which can be bypassed or in contrast — exacerbated in various protocols and adaptations of scRNA-seq, such as single-nuclei RNA-seq and scRNA-seq of formalin-fixed samples which are not discussed here. Broadly speaking, however, limitations of scRNA-seq can be divided into multiple categories depending on the stage of the experiment or data analysis at which they arise (Figure 7).

When discussing issues specific to scRNA-seq, it should be noted that prior to single-cell isolation and RNA extraction, common limitations associated with sample sourcing and preparation resemble those encountered in bulk RNA-seq [111]. When target cells are obtained from a brushing, biopsy or autopsy, parameters such as sites of sampling from a single donor, physiological differences between donors from the same cohort, sample handling and the duration of the period from sampling to sequencing may lead to substantial variability in the results, thereby reducing reproducibility of the experiment and undermining the statistical power. On the other hand, the practice of culture and differentiation of primary cells before sequencing introduces other types of biases since factors ranging from the type of media and plasticware to experimental technique and duration of the experiment can affect both cell type presence and distribution at the point of sampling. For instance, Ruiz García et al. [89] reported that Lonza bronchial epithelial cell growth medium favoured differentiation of MCCs but not goblet cells, whereas the use of PneumaCult-ALI medium resulted in the presence of both cell types in numbers and distributions that were more physiologically relevant. As for the confounders common to samples prepared from any source, the most frequently used cell dissociation protocols involve proteases, which not only can cause cell death of certain cell types or states but can also perturb transcriptomes of profiled cells, leading to the up-regulation of apoptosis- and stress-related genes, e.g. *FOS* and *ATF3* [112]. In bulk RNA-seq this issue can be avoided by bypassing cell dissociation and adding RNA extraction reagents (e.g. TRIzol) directly to the sample or on top of the cell layer, whereas in scRNA-seq, protocol modifications, such as the use of cold active proteases [113], and *in silico* filtering steps have been suggested to mitigate dissociation-induced effects on the gene expression in scRNA-seq.

Technical limitations in commonly used scRNA-seq protocols include cell throughput and transcript coverage by Illumina sequencing (Figure 7A). For example, most of the droplet-based high-throughput scRNA-seq platforms, such as 10× Genomics Chromium, provide almost no information on differential isoform expression as only roughly 100 bp section of the 3′-end of the transcript is sequenced. In addition, it is estimated that only 10–15% of all cellular mRNAs are captured and reverse transcribed in a typical scRNA-seq experiment [65]. Even through variable mRNA capture efficiency can be normalised for with addition of ‘spike-in’ RNA molecules, amounts and other characteristics of which are known [114], this figure implies that only transcripts of the most highly expressed genes are captured. The impact of this can be even more drastic when read depth in terms of genes or UMIs per cell is low. On top of that, the most frequently used technique of RNA selection, oligo-dT enrichment, is 3′-end biased and leaves out non-polyadenylated RNAs, which include short and long non-coding RNAs. Alternative methods for rRNA depletion in scRNA-seq have been reported [115], but none has been widely implemented to date.

Other technical issues in scRNA-seq are also platform-dependent (Figure 7B). For instance, cell doublets — cases when mRNAs from two different cells are captured and tagged with the same barcode within the same droplet, are common in droplet-based protocols such as DROP-seq. They can lead to unique but artificial expression signatures. Doublets can be removed by using strict upper UMI thresholds or applying filters from *in silico* simulations [65]. Another issue for droplet-based protocols is release of RNA molecules from lysed cells and their colocalization within droplets containing single cells. Currently, contaminating RNA molecules can be predicted and filtered out using various *in silico* tools [74,75]. Data from plate-based scRNA methods, in turn, often requires batch correction due to the impact of variability in timing during individual plate processing. Overall, high degree of technical variability in scRNA-seq experiments can hinder direct comparisons and lower the quality of data integration from different platforms. This holds true not only for highly customisable scRNA-seq platforms established in academic settings, but also for commercial ones. To exemplify the latter, scRNA-seq analysis of the human pancreatic islets conducted by Wang et al. [12] on Fluidigm C1 96/HT, Clontech iCell8 and 10× Genomics Chromium showed that both relative fractions of cells from each cell type that was detected, and their DEGs differed significantly between the evaluated platforms.

With Illumina as an NGS platform of choice in scRNA-seq, analysis of generated data is limited by factors, such as ambiguous mapping of short reads, that are applicable to any method involving short-read sequencing [5], but are not discussed here. As for the issues specific to scRNA-seq, it has been reported that the number of genes with expression values of zero caused by technical reasons varies dramatically between the cells and that there are more dropouts in scRNA-seq than expected, particularly among genes with lower expression levels [71] (Figure 7C). For example, Okuda et al. [20] reported that two 3′-end scRNA-seq methods underestimated the number of CFTR-expressing cells among bronchial epithelial cells by roughly a factor of 10.

Apart from technical and sequencing aspects, choice of methods from a wide range of available platform-specific and open-source tools for data analysis steps starting from generation of gene expression count matrices to clustering [72] and trajectory inference [73], may cause differences in results, including cases when data from the same scRNA experiment is re-analysed. Even if a custom end-to-end or default data analysis pipeline is chosen, user input is still required at nearly every stage and can have a substantial impact on the outcomes. For instance, by setting strict threshold values for gene or UMI counts and fraction of mitochondrial reads per cell in order to filter out dead cells during the QC step, novel but rare cell types may be missed [65]. Manual input based on a biological hypothesis or prior knowledge is also crucial for clustering, trajectory inference analyses and annotation of cell types (Figure 7D,E). When unsupervised methods are used or when reference annotations are absent, it is important to first evaluate a range of tools and compare the outcomes to datasets generated using the same protocol, from the same tissue or organism.

## Conclusions and future outlook

On the cusp of technologies capable of elucidating various facets of cellular identity at a resolution of individual cells, scRNA-seq has not only experienced a phase of rapid development, scaling and adoption, but also vividly showcased the true potential of single-cell transcriptomics in the respiratory science and other research fields. Since its inception, scRNA-seq has been front-running the discovery of novel cell types, states and fates by drastically increasing the scope of transcriptome that can be profiled with less bias and more accuracy by eliminating the barrier of averaged gene expression inherent to both microarray analyses and bulk RNA-seq.

Nonetheless, scRNA-seq is not devoid of constraints and challenges. Higher sensitivity makes it susceptible to numerous sources of biological noise that are found in cellular environments, such as transcriptional bursts [40] and oscillations in cell states driven by cell cycle, responses to regular or sporadic internal and external stimuli [116], which create stochastic variation in gene expression between cells that may not be biologically meaningful in the context of a particular study. The extent of true biological variation may also be hidden or skewed by batch and sampling effects, whereas technical sources of variation can arise from different designs of scRNA-seq platforms and custom protocol modifications. In addition, the introduction of frameworks outlining the scope of experimental and data analysis standards as well as the implementation of consensus nomenclature for naming and characterising cell populations discovered with scRNA-seq are lagging behind the technological progress, which makes direct cross-study comparisons and integration of data difficult. Use of reference annotation data for canonical and rare cell types provided by consortia such as HLCA [31,32] may offer a promising way forward, but will still require answers to fundamental questions raised in this review as values for a broad range of parameters, which are likely to be tissue- or organism-specific, need to be determined.

Apart from using reference annotations, the confidence in conclusions drawn about the observed cell types, states and fates on the basis of any scRNA-seq data can be improved by benchmarking the results against the findings of conventional transcriptomic, proteomic and genetic tools. Continuous method development and evaluation for integration and consolidation of scRNA-seq findings will not only facilitate incorporation of data from other emerging single-cell technologies [117,118], including spatial single-cell transcriptomics [64,119] and long-read scRNA-seq for isoform discovery and differential isoform expression analyses [61], but will also pave the way for the construction of truly comprehensive maps of cellular identities.

## Competing Interests

The authors declare that there are no competing interests associated with the manuscript.

## Open Access

Open access for this article was enabled by the participation of University of Sheffield in an all-inclusive *Read & Publish* agreement with Portland Press and the Biochemical Society under a transformative agreement with JISC.

## CRediT Author Contribution

**Colin David Bingle**: Conceptualisation, Supervision, Project administration, Writing — review and editing. **Oleksandr Dudchenko**: Conceptualisation, Formal analysis, Investigation, Writing — original draft, Writing — review and editing. **Jose Ordovoaes-Montanes**: Writing — original draft, Writing — review and editing.

## Abbreviations

ALI: air–liquid interface
cDNA: complementary DNA
CF: cystic fibrosis
CFTR: cystic fibrosis trans-membrane conductance regulator
COVID-19: coronavirus disease 2019
DEGs: differentially expressed genes
HBECs: human bronchial epithelial cells
HLCA: human lung cell atlas
MCCs: multiciliated cells
NGS: next generation sequencing
SARS-CoV-2: severe acute respiratory syndrome coronavirus clade 2
scRNA-seq: single-cell RNA-sequencing
UMI: unique molecular identifier
